# Auditory Motion Information Drives Visual Motion Perception

**DOI:** 10.1371/journal.pone.0017499

**Published:** 2011-03-09

**Authors:** Souta Hidaka, Wataru Teramoto, Yoichi Sugita, Yuko Manaka, Shuichi Sakamoto, Yôiti Suzuki

**Affiliations:** 1 Department of Psychology, Rikkyo University, Niiza-shi, Saitama, Japan; 2 Department of Psychology, Graduate School of Arts and Letters, Tohoku University, Sendai, Miyagi, Japan; 3 Research Institute of Electrical Communication, Tohoku University, Sendai, Miyagi, Japan; 4 Neuroscience Research Institute, National Institute of Advanced Industrial Science and Technology (AIST), Tsukuba, Ibaraki, Japan; 5 Core Research for Evolutional Science and Technology (CREST), Japan Science and Technology Agency, Tokyo, Japan; Claremont Colleges, United States of America

## Abstract

**Background:**

Vision provides the most salient information with regard to the stimulus motion. However, it has recently been demonstrated that static visual stimuli are perceived as moving laterally by alternating left-right sound sources. The underlying mechanism of this phenomenon remains unclear; it has not yet been determined whether auditory motion signals, rather than auditory positional signals, can directly contribute to visual motion perception.

**Methodology/Principal Findings:**

Static visual flashes were presented at retinal locations outside the fovea together with a lateral auditory motion provided by a virtual stereo noise source smoothly shifting in the horizontal plane. The flash appeared to move by means of the auditory motion when the spatiotemporal position of the flashes was in the middle of the auditory motion trajectory. Furthermore, the lateral auditory motion altered visual motion perception in a global motion display where different localized motion signals of multiple visual stimuli were combined to produce a coherent visual motion perception.

**Conclusions/Significance:**

These findings suggest there exist direct interactions between auditory and visual motion signals, and that there might be common neural substrates for auditory and visual motion processing.

## Introduction

The primate brain effectively associates or integrates information from different modalities in order to establish robust representations of the outer world [1], [2]. It has been considered that multisensory information processing is more closely related and mutually interactive than classical views had assumed [3]. With regard to audiovisual interaction in motion perception, the effect of visual motion information on auditory motion perception has been mainly reported. For example, the adaptation made in response to moving visual stimuli induces a motion aftereffect in the auditory modality [4]. Moving visual stimuli also capture the perceived motion direction of the auditory stimulus [5]–[7]. These findings suggest that there are common neural substrates to motion perception between the visual and auditory modalities [8].

The modulatory effect of auditory information on visual motion perception has been also reported. A transient sound disambiguates bistable visual motion perception [9], [10] by capturing the temporal positional information of a moving visual stimulus [11]. However, the inducing or driving effect of auditory information had not yet been reported. The effect of auditory motion information on visual motion direction perception was found to be absent [6], very weak [12], or indistinguishable from a response bias [13].

The lack of an inducing or driving effect of auditory motion information on visual motion perception was interpreted based on the reliability-based concept of multimodal interaction [1]; visual systems are usually superior to auditory systems in spatial processing so that auditory information has no effect on vision in motion processing [7]. However, it has recently been demonstrated that the alternation of sound location can induce illusory visual motion perception to a static stimulus [14], [15]. This phenomenon is called as sound-induced visual motion (SIVM). In SIVM, a blinking visual stimulus at a fixed location was perceived to be laterally moving when it was synchronized with an alternating left-right sound source. SIVM was clearly observed when the visual stimulus was presented in the peripheral visual field (more than 10 deg). In line with the study on spatial localization [16] and the reliability-based concept [1], the findings regarding SIVM suggest that auditory information becomes relatively more reliable in motion perception when visual information is vulnerable or degraded in the peripheral visual field.

Whereas SIVM provides strong evidence demonstrating the inducing or driving effect of auditory information on visual motion perception, the underlying mechanisms are still unclear. It is possible that an alternating left-right sound captures the positions of the visual stimuli, resulting in visual motion perception (auditory positional capture) [16], [17]. It is also possible that the auditory motion signals could directly contribute to visual motion perception, for example, when an alternating left-right sound source is perceived to be moving (i.e., apparent motion) [18], the moving sound source directly triggers motion perception of a static stimulus (c.f. *visual* motion capture) [19], [20].

**Figure 1:**
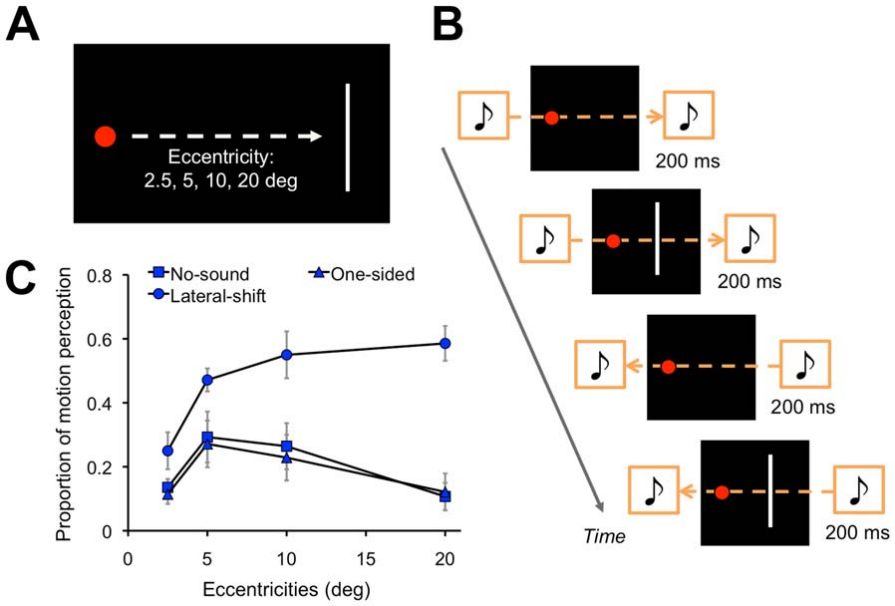
Schematic illustrations of the experimental design and results of Experiment 1. (A) Visual stimuli. (B) Time course of the presentation of auditory and visual stimuli. (C) Results. The vertical axis denotes the proportion of motion perception to the static visual stimuli. The horizontal axis denotes the retinal eccentricities of the visual stimuli. The error bars denote the standard error of the means.

The aim of the current study was to investigate the direct contributions of auditory motion signals on visual motion perception. In the first experiment (Experiment 1), we investigated how SIVM occurred in a situation where the spatiotemporal position of the flashes was located in the middle of the trajectory of a lateral auditory motion provided by a virtual stereo noise source smoothly shifting in a horizontal plane (either left to right or right to left) (Figure 1B). In this situation, the lateral position of the sound gradually changes from side to side, so that we can present a flash at the moment the sound is located near the flash in lateral position. Because there was little discrepancy in lateral position between the sound and flash, auditory positional information would have little influence on the perceived position of visual stimuli. If SIVM was observed in this situation, we could assume that auditory motion information directly contributes to visual motion perception.

**Figure 2:**
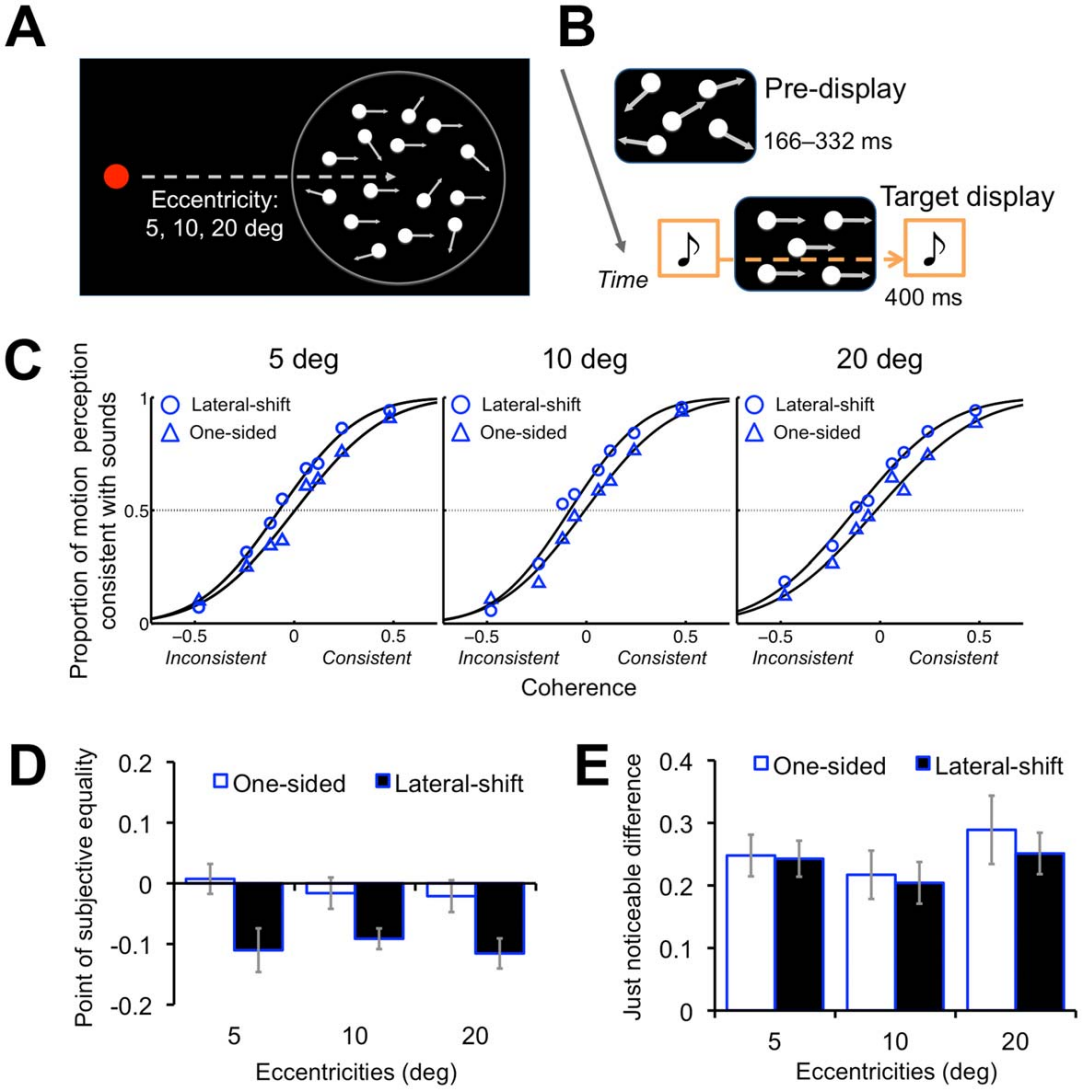
Schematic illustrations of the experimental design and results of Experiment 2. (A) Global motion display containing multiple local motion vectors. (B) Time course of the presentation of auditory and visual stimuli. (C) Psychometric curves as the proportion of motion direction perception consistent with sounds against the motion coherence. In the horizontal axis, the positive values indicate the situation where visual motion direction and alternate direction of sound (lateral-shift condition) or the presented location (one-sided condition) of the sounds was consistent. The inconsistent situation is represented by negative values. (D) The point of subjective equality (PSE) and (E) Slope of psychometric functions (JND) obtained in each retinal eccentricity. The error bars indicate the standard error of the means.

The second experiment (Experiment 2) further investigated the effect of an auditory motion signal on visual motion perception without one-to-one correspondence between the auditory and visual stimuli. Together with the lateral auditory motion, we presented a global visual motion display in which different localized motion signals, contained in multiple visual stimuli, were combined to produce a coherent motion perception [21] (Figure 2A). There was no clear one-to-one correspondence between the auditory stimuli and each visual stimulus. If the auditory stimuli containing motion information affected integrated visual motion information and its perception, this would provide strong evidence for a direct interaction between auditory and visual modalities in motion processing.

## Methods

### Ethics Statement

Written consent was obtained from each participant prior to experiments. The experiments were approved by the local ethics committee of Graduate School of Arts and Letters at Tohoku University.

### Participants and Apparatus

Seven volunteers participated in Experiments 1 and 2; both experimental groups included two of the authors (S.H. and W.T.). All participants were experienced observers in psychophysical experiments, and five participants were naive to the purpose of the experiments. All participants had normal or corrected-to-normal vision and normal hearing. We presented the visual stimuli on a CRT display (Sony Trinitron GDM-FW900, 24 inch) with a resolution of 1600×1200 pixels and a refresh rate of 60 Hz. Sounds were presented through an audio interface (Roland EDIROL FA-66) and headphones (Sennheiser HDA 200). A customized PC (Dell-Dimension 8250) and MATLAB (The Mathworks, Inc.) with the Psychophysics Toolbox [22], [23] were used to control the experiments. We confirmed that the onset of the visual and auditory stimuli was synchronized using a digital oscilloscope (Iwatsu TS-80600). All of the experiments were conducted in a dark room.

### Stimuli

#### Experiment 1

A red circle (0.4 deg in diameter; 17.47 cd/m^2^) was presented as a fixation point on a black background (0.4 cd/m^2^). A sequence of white bars (0.2 deg×3 deg; 4.99 cd/m^2^) was presented as visual stimuli at an eccentricity of either 2.5, 5, 10, or 20 deg along the horizontal plane (Figure 1A). A white noise was presented as an auditory stimulus for 400 ms with a cosine ramp of 5 ms at the onset and offset. The sampling frequency was 22050 Hz. The white noise was created per trial. There were three sound conditions: lateral-shift, one-sided, and no-sound. The lateral smooth movement of a virtual sound source was generated by cross-fading a pair of two white noises of 400 ms duration between the left and right ears (lateral-shift condition). For the cross-fading, each white noise was initially presented at the sound pressure level (SPL) of 85 dB and was then faded to null. The cross-fading white noise was presented two times without interstimulus interval (ISI) so that a virtual sound source was simulated to move from one side to the other and return to the original side, while the total sound power was kept constant. In the one-sided condition, a sound with a constant SPL of 85 dB (400 ms duration) was presented two times either to the left or right ears without ISI. In these conditions, the visual stimulus was presented for 200 ms in the middle of the cross-fading sound (lateral-shift condition) or the constant (one-sided condition) sound with 200 ms of ISI (Figure 1B). In each trial of these conditions, each white noise was presented 6 times and the visual stimulus was presented 5 times in total. In the no-sound condition, only the visual stimulus was presented 5 times. Each trial began with the presentation of the fixation point for 500 ms. The visual stimuli were presented in the participant's dominant eye field.

#### Experiment 2

A global motion display containing 200 white (4.99 cd/m^2^) dots was presented as visual stimuli. The diameter of each dot was 0.1 deg, and each dot was randomly located within 5 deg in diameter of an invisible circular window (Figure 2A). The global motion display was presented at an eccentricity of either 5, 10, or 20 deg. At the beginning of each trial, each dot moved in a random direction from 0 to 360 deg for 166 ms (10 frames) to 332 ms (20 frames). Then, the target motion signal was presented for 400 ms (24 frames), during which 6, 12, 24, or 48% of the dots moved either 0 deg (left) or 180 deg (right) as a target direction (Figure 2B). The other dots moved in a random direction except for the target motion direction. The lifetime of each dot was 2 frames. The velocity of each dot was 8 deg/s. The lateral-shift and one-sided conditions were both tested. The cross-fading sound or the constant sound was presented for 400 ms with 0 ms of ISI. The onset of the sounds was synchronized with that of the target motion signal (Figure 2B). Except for these variations, the stimulus parameters were identical to those of Experiment 1.

### Procedure

#### Experiment 1

We asked the participants to place their heads on a chin rest, fixate on a red circle, and judge whether a flash (white bar) was perceived to be moving or not. This experiment consisted of a training session and a main experimental session. In the training session, the participants were asked to discriminate between static and moving visual stimuli for 80 trials: visual stimuli (2: static/moving) × eccentricities (4) × repetitions (10). The white bar was displaced back and forth by 0.2 deg in the horizontal direction when it moved. The training session was repeated until the discrimination performance reached above 75% for each eccentricity. The main session consisted of 240 trials where visual stimuli were always static: eccentricities (4) × auditory stimuli (3) × repetitions (20). In the lateral-shift condition, the first sound was delivered to the right ear for one-half of the trials and to the left ear for the other half. In the one-sided condition, the sounds were delivered to the right ear for one-half of the trials and to the left ear for the other half. The presentation order of the conditions and the location of the first sound (left/right) were randomized in the main session. Additionally, 96 filler trials where the white bar was actually displaced by 0.2 deg in the horizontal direction were randomly introduced in the trials of the main session: eccentricities (4) × auditory stimuli (3) × repetitions (8). In the filler trials, the white bar was physically displaced to the right for the rightward auditory motion and to the left for the leftward auditory motion because our preliminary observation confirmed that the perceived motion direction was congruent between the auditory and visual stimuli in the lateral-shift condition. In the one-sided condition, the initial onset position was consistent between the auditory and visual stimuli. The initial onset position of the visual stimuli (and that of the sounds) was randomized in the lateral-shift and one-sided conditions, and was randomly assigned in the no-sound condition.

#### Experiment 2

The participants were asked to fixate on a red circle and to report the perceived visual motion direction (left or right). This experiment contained only the main session. The main session consisted of 960 trials: auditory conditions (2: lateral-shift/one-sided) × auditory motion direction or location (2: left (ward) /right (ward)) × visual target motion direction (2: leftward/rightward) × coherence (4: 6, 12, 24, and 48%) × eccentricities (3: 5, 10, and 20 deg) × repetitions (10). The motion direction of the visual target and the auditory stimuli (lateral-shift condition) or the presented location (one-sided condition) of the sounds was either consistent or inconsistent. For example, leftward visual motion was presented with leftward (lateral-shift condition) or left-sided (one-sided condition) auditory stimulus in the consistent situation, while leftward visual motion with rightward (lateral-shift condition) or right-sided (one-sided condition) auditory stimulus in the inconsistent situation). The presentation order of the conditions and the target motion directions were randomized.

## Results

We confirmed that the data without the authors' responses and those including them were not statistically different (see Figure S1). Thus, we included the authors' data in later analyses.

### Experiment 1

The proportion of motion perception to the static visual stimuli was calculated (Figure 1C). Then, we conducted a repeated measures analysis of variance (ANOVA) with 2 within-participant factors: eccentricities (2.5, 5, 10, and 20 deg) and auditory conditions (lateral-shift, one-sided, and no-sound). The ANOVA revealed a significant main effect of eccentricities (*F*_3, 18_ = 5.87, *p*<.01) and auditory conditions (*F*_2, 12_ = 19.54, *p*<.001). An interaction effect between these factors was also significant (*F*_6, 36_ = 7.16, *p*<.001). Regarding the significant simple main effect of auditory conditions (5 deg: *F*_2, 48_ = 5.78, *p*<.01; 10 deg: *F*_2, 48_ = 14.88, *p*<.001; 20 deg: *F*_2, 48_ = 35.54, *p*<.001), the post-hoc test (Tukey's HSD, *p*<.05) revealed that the proportion of motion perception was higher in the lateral-shift condition than the other conditions for 5, 10, and 20 deg of eccentricity. In contrast, there were no significant differences between the one-sided and no-sound conditions.

The results of Experiment 1 clearly showed that the lateral auditory motion of a single sound image smoothly shifting in the horizontal plane induced motion perception to the static visual stimulus when the spatiotemporal position of the visual stimuli was in the middle of the auditory motion trajectory; the flashes at a fixed location appeared to be moving laterally in the lateral-shift condition. This effect was clearly observed at the eccentricities larger than parafovea (5, 10, and 20 deg), and the effect appeared to become obvious as retinal eccentricities increased. This indicates that the degraded reliability of the visual stimuli increased the effect of sounds on visual motion perception [14], [15]. In contrast, the absence of a significant effect of sound in the one-sided condition suggested that the results in the lateral-shift condition were inattributable to the effect of the sound presentation itself.

**Figure 3:**
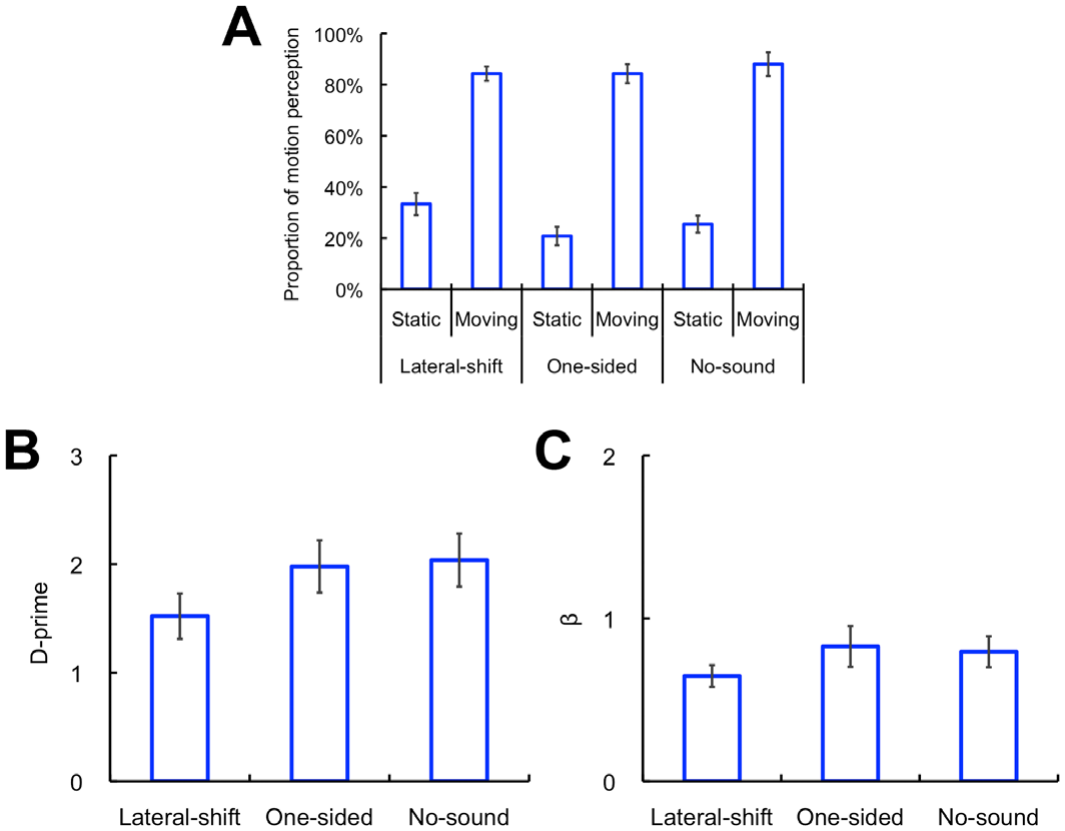
D-prime data. (A) Proportion of motion perception, (B) d-primes, and (C) response criterion (β). The horizontal axis denotes the types of auditory and visual conditions. The error bars denote the standard error of the means.

It would certainly be possible that the moving sound might induce response or decisional bias; the participants might expect that they perceived visual stimuli to move whenever the moving sound was presented. Thus, we conducted an additional experiment in order to test this possibility. The lateral-shift, one-sided, and no-sound conditions were presented as the auditory stimuli. The visual stimuli were either static or moving (0.2 deg) and presented at 10 deg of retinal eccentricity. Each block consisted of 144 trials (auditory conditions (3) × visual conditions (2) × repetitions (24)). To reduce the bias to the maximum possible degree, all of the participants (N = 10) were newly recruited and naïve to the purpose of the experiment; moreover, we provided them visual feedback (words such as “Correct!”) to inform whether or not their judgments were correct in each trial. Except for these variations, the stimulus parameters and procedures were identical to those in Experiment 1. On the basis of the proportional data, we calculated d-prime as the index of sensitivity, which can be separated from the index of criterion or response bias (β) [24] (Figure 3). The responses of perceiving static stimuli were regarded as a “hit” for the static trials and as a “false alarm” for the moving trials in the visual conditions. With regard to d-prime, a one-way repeated measures ANOVA revealed a significant main effect of the auditory conditions (*F*_2, 18_ = 4.49, *p*<.05). A post-hoc test (Tukey's HSD, *p*<.05) revealed that the d-prime value in the lateral-shift condition was smaller than that in the other conditions. In contrast, there was no significant main effect regarding β (*F*_2, 18_ = 1.67 *p* = .22). When the visual stimulus was in motion, the participants correctly perceived the motion in all of the conditions. In contrast, the static visual stimulus was illusorily perceived as moving only when the sounds traveled in the lateral direction. These phenomenal aspects were clearly shown in the decrement of d-prime in the lateral-shift condition. We further confirmed that the effect of the lateral shifts in sounds was so strong that the participants could not distinguish between actual and illusory motion even if they were given feedback, and that the changes in sensitivity (d-prime) were independent from those in criterion (β). These results suggest that the continuous left-right shifts of the virtual sound source actually changed the sensitivity to visual motion perception, and that the findings in Experiment 1 could not be simply explained by the response or the decisional bias.

**Figure 4:**
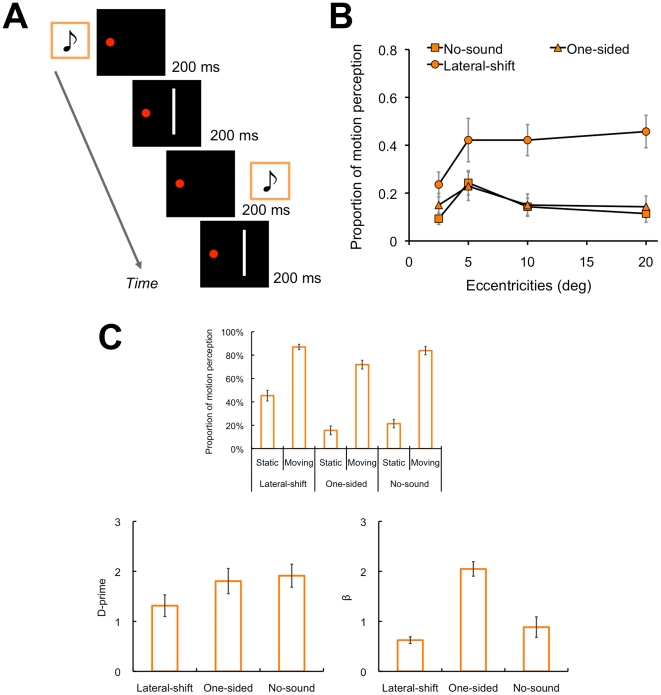
Data for discrete shifts of sound source. (A) Time course of the presentation of auditory and visual stimuli. The horizontal shift of a sound (lateral-shift condition) was demonstrated by presenting the sound alternately to the left and right ears. In the one-sided condition, the sound was presented to either the left or the right ear. In these conditions, the sound was presented 6 times for 200 ms each with 200 ms of ISI, and the visual stimulus of 200 ms in duration was presented in between 2 successive sounds; that is, the visual stimulus was presented 5 times with 200 ms of ISI. In the no-sound condition, only the visual stimulus was presented 5 times. Except for these sound manipulations, the stimulus parameters and procedures were consistent with those of Experiment 1. (B) Results (N = 7). The participants were the same as those of Experiment 1. The vertical axis denotes the proportion of motion perception to the static visual stimuli. The horizontal axis denotes the retinal eccentricities of the visual stimuli. The error bars denote the standard error of the means. A repeated measures analysis of variance (ANOVA) with eccentricities (2.5, 5, 10, and 20 deg) and auditory conditions (lateral-shift, one-sided, and no-sound) revealed a significant main effect of auditory conditions (*F*_2, 12_ = 23.03, *p*<.001). An interaction effect between these factors was also significant (*F*_6, 36_ = 2.56, *p*<.05). Regarding the significant simple main effect of the auditory conditions (5 deg: *F*_2, 48_ = 9.70, *p*<.001; 10 deg: *F*_2, 48_ = 14.03, *p*<.001; 20 deg: *F*_2, 48_ = 18.83, *p*<.001), the post-hoc test (Tukey's HSD, *p*<.05) revealed that the proportion of motion perception was higher in the lateral-shift condition than the other conditions for 5, 10, and 20 deg of eccentricity. (C) D-prime data (N = 8, all of which were newly recruited naïve participants). In another experiment, we estimated d-prime and β values for the discrete sounds (see the section of Experiment 1 in the Results part for details). With regard to d-prime, a one-way repeated measures ANOVA revealed a significant main effect of the auditory conditions (*F*_2, 14_ = 8.14, *p*<.005). The post-hoc test (Tukey's HSD, *p*<.05) revealed that the d-prime value in the lateral-shift condition was smaller than that in the other conditions. In contrast, the β value in the one-sided condition was higher than that in the other conditions (ANOVA: *F*_2, 14_ = 9.08, *p*<.005; post-hoc test: *p*<.05). This tendency was inconsistent with that of d-prime so that the changes in sensitivity could be assumed to be independent from those in criterion.

The results of Experiment 1 demonstrated that the SIVM occurred even when auditory laterality information could have little influence on the perceived position of visual stimuli. We also confirmed that this was true even for discrete shifts of sound and the resulting auditory apparent motion [18], which was used in the previous studies [14], [15] (Figure 4). The mechanism underlying the SIVM would be the direct interaction between the motion signal contained in the lateral shifts in sounds (leftward or rightward) and visual motion perception rather than the auditory capture on visual localization [16], [17].

### Experiment 2

We depicted psychometric curves as the proportion of visual motion direction perception consistent with the lateral shifts in sounds as a function of the visual motion coherence in each eccentricity and each auditory condition (Figure 2C). Then, we estimated the point of subjective equality (PSE) as the 50% threshold of the psychometric functions by fitting a cumulative Gaussian distribution function to each participant's data using a maximum likelihood method (Figure 2D). A repeated measures ANOVA with 2 within-participant factors, auditory conditions (lateral-shift, one-sided) and eccentricities (5, 10, and 20 deg), was conducted. This revealed that the main effect of the auditory conditions was significant (*F*_1, 6_ = 7.52, *p*<.05); the PSE in the lateral-shift condition shifted toward the inconsistent direction as compared to that in the one-sided condition. The main effect of the eccentricities (*F*_2, 12_ = .53, *p* = .60) and the interaction between the factors (*F*_2, 12_ = 1.11, *p* = .36) were not significant.

The results showed that the lateral auditory motion provided by smooth shifts of a single sound image in horizontal plane altered the motion direction perception of a global visual motion signal. Specifically, the lateral auditory motion perceptually cancelled out the opposite lateral visual motion information and induced consistent motion perception to the visual stimuli. Contrary to the previous studies [12], [13], the current results clearly demonstrate that continuous lateral shifts of sound can induce visual motion direction perception consistent with auditory movement in a global motion display.

With respect to the effect of an auditory motion signal on a global visual motion display, it was reported that auditory motion information affected the judgments of perceived visual motion direction only when the coherence of the visual local motion signal was considerably low [13]. This result indicates that auditory motion information was utilized for making decisions only when the visual motion direction was ambiguous and hard to discriminate alone. If this decisional biasing effect existed in Experiment 2, the slope of the psychometric functions would become less steep especially at the center against the visual stimuli with lower coherences. In order to confirm this possibility, we calculated the slope of psychometric functions as just noticeable differences (JND) by the following formula: (75% threshold – 25% threshold)/2 (Figure 2E). A repeated measures ANOVA revealed that the main effect of the auditory conditions (*F*_1, 6_ = 2.16, *p* = .19), that of the eccentricities (*F*_2, 12_ = 1.67, *p* = .23), and the interaction between these factors (*F*_2, 12_ = 1.11, *p* = .36) were not significant. We confirmed that the slopes of each psychometric function were consistent between the auditory conditions, indicating that the decisional criteria of motion direction perception were consistent between the auditory conditions. We, therefore, could consider that the decisional bias was unattributable to the main factor.

The results of Experiment 2 indicate that the auditory motion information contained in continuous lateral shifts of a sound image can directly alter visual motion perception extracted from different localized motion vectors of multiple visual stimuli. Therefore, we could consider that motion processing and perception directly interact between auditory and visual modalities.

## Discussion

We found that a lateral auditory motion provided by a pair of cross-fading white noises smoothly shifting along a horizontal trajectory induced illusory visual motion perception (SIVM) even when the flash was presented in the middle of the trajectory of the sound shifts; the spatial position of the virtual sound source was perceived around the visual stimulus at the moment the flash was presented, and the laterality information of the sound (left or right) could have little influence on the visual stimuli (Experiment 1). It was also revealed that the lateral auditory motion altered the visual motion direction perception in a global motion display (Experiment 2); different localized motion signals of multiple visual stimuli were combined to produce a coherent visual motion perception so that one-to-one correspondence between the auditory and visual stimuli was hard to be established. These findings suggest that there exists direct audio-visual interaction in motion processing, and that there might be common neural substrates for auditory and visual motion processing.

Eye movements might be induced by the left-right shifts of sound. However, we confirmed that the SIVM occurred without eye movements (see Figure S2). In Experiment 2, the lifetime of each dot in our global motion display was only two frames, making it difficult to associate eye movements with each visual stimulus. We, therefore, could conclude that eye movements did not play a decisive role in the results of the present study.

The involvement of response or decisional bias might be also suspected. However, in Experiment 1, we found that the d-prime in the lateral-shift condition was lower than that in the other conditions, whereas the β values did not differ among the conditions (Figure 3; see also Figure 4C). These results suggest that the left-right shifts of virtual sound source indeed change the sensitivity of motion perception in SIVM. Moreover, we also confirmed that the JNDs for global visual motion display were consistent between the auditory conditions in Experiment 2; instead, the PSEs changed by the lateral auditory motion generated by continuous left-right shifts of the virtual sound source (Figure 2D and E). This means that the auditory motion signal could perceptually cancel out the opposite visual motion signal and induce consistent motion perception to the visual stimuli in almost all of the coherences, namely even when there were relatively sufficient visual motion signals. These results indicate that the current findings could not be simply explained by the biases.

Based on these findings, we can consider that the audio-visual interaction involved in motion processing could explain the current phenomenon. In the previous studies [14], [15], two possible mechanisms were considered regarding the auditory inducing effect on visual motion perception. One was the direct interaction of motion information between the auditory and visual modalities (c.f. *visual* motion capture) [19], [20]. Another mechanism is auditory capture on visual localization [16], [17] in which the auditory spatial information (left or right) simply modulates visual inputs in a spatial domain. The current research demonstrated that SIVM occurred even when auditory laterality information could have little influence on the perceived position of visual stimuli. We also found that the continuous shifts of the virtual sound source altered the perception of global visual motion where there was no clear correspondence between the auditory stimuli and each of the visual stimuli. These findings indicate that auditory motion information can directly trigger or induce visual motion perception.

Some previous studies showed that auditory motion affected visual motion perception. For example, auditory motion could direct an ambiguous, bistable motion perception to an unambiguous one [25]. This finding indicates that auditory motion information could modulate visual motion perception. In contrast, our current findings demonstrated that auditory motion information triggered motion perception to static visual stimuli and drove motion perception against global visual motion signals. It was also reported that auditory motion affected the perception of a global visual motion display [13]. However, this effect could be primarily explained by response or decisional bias because the auditory effect was dominant only when the visual motion signal was highly ambiguous [12], [25]. On the contrary, as mentioned above, our results showed that auditory motion information affected global visual motion perception even when the visual signal contained highly coherent motion signals, and this effect could be distinguishable from response bias. We, therefore, could consider that the current findings are unique in that they demonstrate the driving and inducing effects of auditory motion on visual motion perception.

In Experiment 1, the effect of eccentricity was observed; SIVM frequently occurred at the eccentricities larger than parafovea (5, 10, and 20 deg), and the effect of auditory motion appeared to become more obvious with increasing retinal eccentricities (Figure 1C). In line with the previous study [14], [15], we could assume that the auditory effect became obvious when the visibility or reliability of visual inputs degraded in the peripheral vision. This reliability-based theory would be consistent with the concept regarding the manner in which multimodal integration occurs [1]. In contrast, the effect of the eccentricities seemed not to be observed in Experiment 2; the effect of auditory motion was almost identical among the eccentricities (Figure 2D). The global motion display consisted of multiple visual stimuli with different localized motion signals. In the experiment, the coherence of the motion varied from trial to trial. Moreover, since the lifetime of each stimulus was only 2 frames, it was hard to discriminate or identify each stimulus. Since these manipulations alone could considerably degrade the reliability of the visual stimuli, the left-right sound source would induce the robust auditory effect on visual motion information among the parafoveal and peripheral visual fields.

Contrary to the current research, the previous studies showed that the effect of auditory motion information on visual motion perception was not obvious [12] or indistinguishable from biases [13] in a global visual motion display. The discrepancy might be considered in terms of the eccentricity of visual stimuli. In a previous study [13], the fixation point was presented at the center of the participants' global motion display (16 deg ×16 deg) so that the participants received the visual information from both the left and right visual field, including the fovea and parafovea (±8 deg). In another study [12], participants were presented with relatively large stimuli (50 deg ×38 deg) as a global motion display. However, a fixation point was not presented so that the participants could scan the visual stimuli with their eye movements during the presentation (670 ms). On the contrary, our global motion display was relatively small (5 deg in diameter) and was presented only in the participants' dominant eye field together with the fixation point. In the previous studies, therefore, the visibility or reliability of the visual stimuli might be kept higher than that of the auditory stimuli so that the effect of auditory motion on visual motion perception would not be manifested. It was also notable that whereas the previous studies presented auditory stimuli through loud speakers, we used headphones for the presentation of sounds. Thus, the factor of spatial co-localization between the visual and auditory stimuli [26] also might be different between the previous and current studies. A detailed investigation regarding these issues is beyond the purposes of the current research and should be addressed in future research.

In summary, the current study demonstrates that auditory motion signals can drive or induce visual motion perception consistent with auditory motion perception. The effect of auditory motion signals becomes obvious when the reliability of visual inputs is degraded. We have confirmed that the current results were not explained by auditory position capture effect, eye movements, or biases. The evidence of our study suggests the existence of direct interactions and common neural substrates between the auditory and visual modalities in motion processing and motion perception.

## Supporting Information

Figure S1 **Data with and without the authors' responses.** In order to compare the data without the authors' responses and those including them, we conducted a mixed-design ANOVA by adding the factor of authors (2; with/without the authors' data) as a between-subjects variable to main analyses (with regard to the main analyses, see the Result part for details) (A) Experiment 1. A main effect and interaction effects related to the factor of authors were not significant for continuous (authors: *F*_1, 10_ = .08, *p* = .79; authors×auditory conditions: *F*_2, 20_ = .44, *p* = .65; authors×eccentricities: *F*_3, 30_ = .44, *p* = .94; authors×auditory conditions×eccentricities: *F*_6, 60_ = .18, *p* = .98) and discrete (authors: *F*_1, 10_ = .09, *p* = .77; authors×auditory conditions: *F*_2, 20_ = .36, *p* = .71; authors×eccentricities: *F*_3, 30_ = .16, *p* = .93; authors×auditory conditions×eccentricities: *F*_6, 60_ = .17, *p* = .98) shifts of sound source. (B) Experiment 2. A main effect and interaction effects related to the factor of authors were not significant for point of subjective equality (authors: *F*_1, 10_ = .47, *p* = .51; authors × auditory conditions: *F*_1, 10_ = .11, *p* = .75; authors × eccentricities: *F*_2, 20_ = .36, *p* = .70; authors × auditory conditions × eccentricities: *F*_2, 20_ = .07, *p* = .93) and just noticeable difference (authors: *F*_1, 10_ = .70, *p* = .42; authors×auditory conditions: *F*_1, 10_ = .65, *p* = .44; authors × eccentricities: *F*_2, 20_ = .19, *p* = .89; authors × auditory conditions×eccentricities: *F*_2, 20_ = .01, *p* = .99).
(TIF)

Figure S2 **Eye movement data.** We conducted a control experiment of Experiment 1 in which eye movements were recorded (continuous block). We also collected data for the discrete sounds (discrete block) (see Figure 4). The lateral-shift and no-sound conditions were presented as the auditory stimuli. The visual stimuli were presented at 10 deg of retinal eccentricity. Each block consisted of 80 trials of the main session with a static flash (auditory conditions (2)×repetitions (40)) and 32 trials of the filler session with a moving (0.2 deg) flash (auditory conditions (2)×repetitions (16)). The participant's eye position was recorded from the left eye at a sampling rate of 60 Hz with EMR-9 (NAC Image Technology, Inc.). Except for these variations, the stimulus parameters and procedures were identical to those of Experiment 1 or the additional experiment for discrete sounds. Trials in which eye position deviated by more than 1 deg of visual angle in the horizontal direction from the center of the fixation point during the stimulus presentation were discarded from the analysis. Whereas 12.1±4.8 (SEM) % and 9.2±3.0 (SEM) % of trials were excluded in the continuous block, 12.1±5.6 (SEM) % and 21.1±6.4 (SEM) % of trials were excluded in the discrete block in each auditory condition (lateral-shift and no-sound), respectively. (A) Proportion of visual motion perception without eye movements (N = 6, including 2 of the authors (S.H. and W.T.)). The error bars denote the standard error of the means. A paired two-tailed *t* test confirmed that the reliable amount of motion perception occurred in the lateral-shift condition in each block (continuous block: *t*(5) = 5.25, *p*<.005; discrete block: *t*(5) = 3.11, *p*<.05). We, therefore, could assume that eye movement was not a decisive factor of the result for SIVM. (B) Examples of eye movement recording data for a participant. The upper and lower data show the time course of eye position for the lateral-shift and no-sound conditions in each block, respectively. The data for all trials are shown, except for those in which the eye deviation was more than 1 deg.
(TIF)
