# Supplementary figures and images for: Auditory Motion Information Drives Visual Motion Perception
Source: PLoS One. 2011 Mar 9;6(3):e17499. doi: 10.1371/journal.pone.0017499 (PMC3052321; doi:10.1371/journal.pone.0017499)

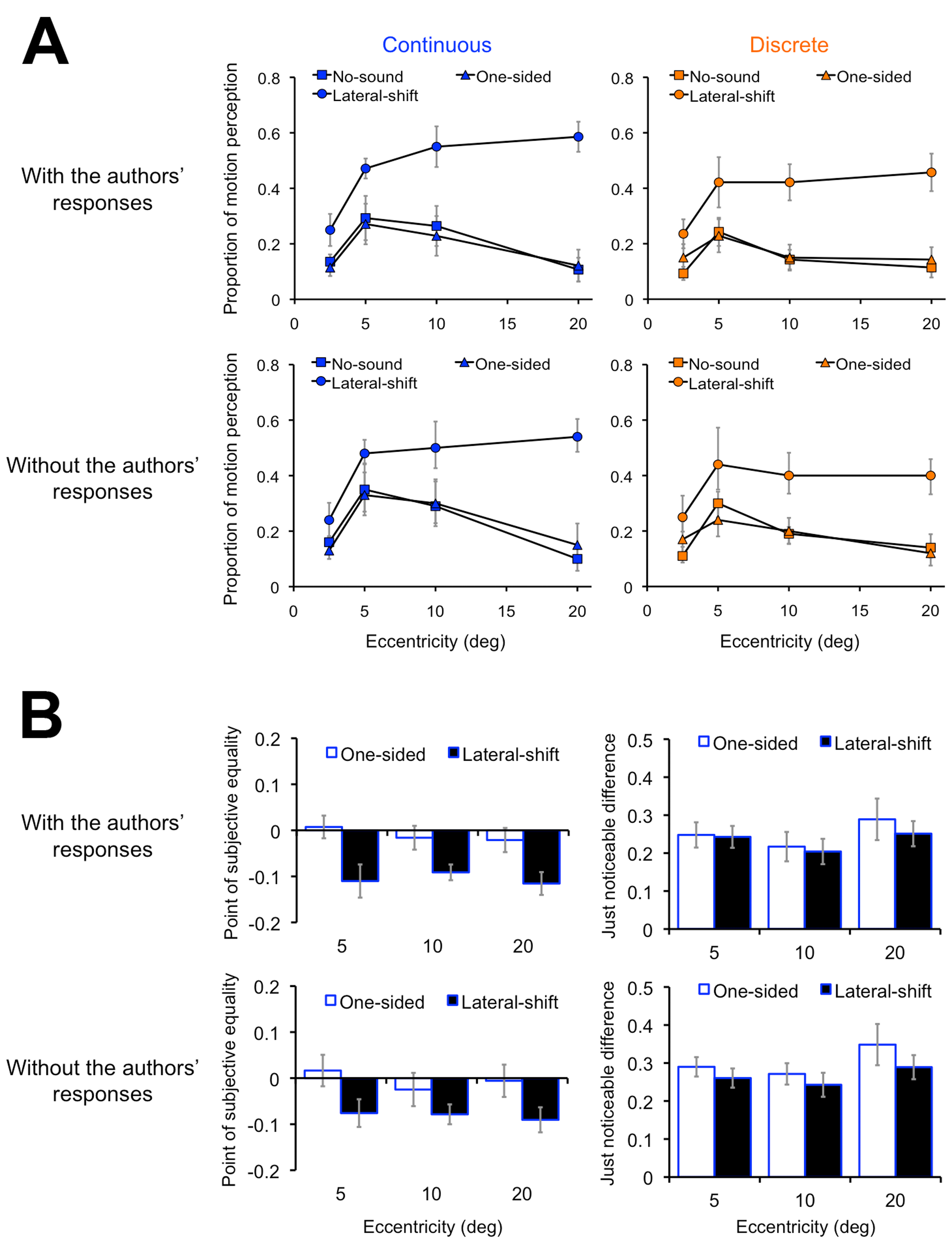

Supplement: Figure S1 — Data with and without the authors' responses. In order to compare the data without the authors' responses and those including them, we conducted a mixed-design ANOVA by adding the factor of authors (2; with/without the authors' data) as a between-subjects variable to main analyses (with regard to the main analyses, see the Result part for details) (A) Experiment 1. A main effect and interaction effects related to the factor of authors were not significant for continuous (authors: F 1, 10 = .08, p = .79; authors×auditory conditions: F 2, 20 = .44, p = .65; authors×eccentricities: F 3, 30 = .44, p = .94; authors×auditory conditions×eccentricities: F 6, 60 = .18, p = .98) and discrete (authors: F 1, 10 = .09, p = .77; authors×auditory conditions: F 2, 20 = .36, p = .71; authors×eccentricities: F 3, 30 = .16, p = .93; authors×auditory conditions×eccentricities: F 6, 60 = .17, p = .98) shifts of sound source. (B) Experiment 2. A main effect and interaction effects related to the factor of authors were not significant for point of subjective equality (authors: F 1, 10 = .47, p = .51; authors × auditory conditions: F 1, 10 = .11, p = .75; authors × eccentricities: F 2, 20 = .36, p = .70; authors × auditory conditions × eccentricities: F 2, 20 = .07, p = .93) and just noticeable difference (authors: F 1, 10 = .70, p = .42; authors×auditory conditions: F 1, 10 = .65, p = .44; authors × eccentricities: F 2, 20 = .19, p = .89; authors × auditory conditions×eccentricities: F 2, 20 = .01, p = .99). (TIF) [file pone.0017499.s001.tif]

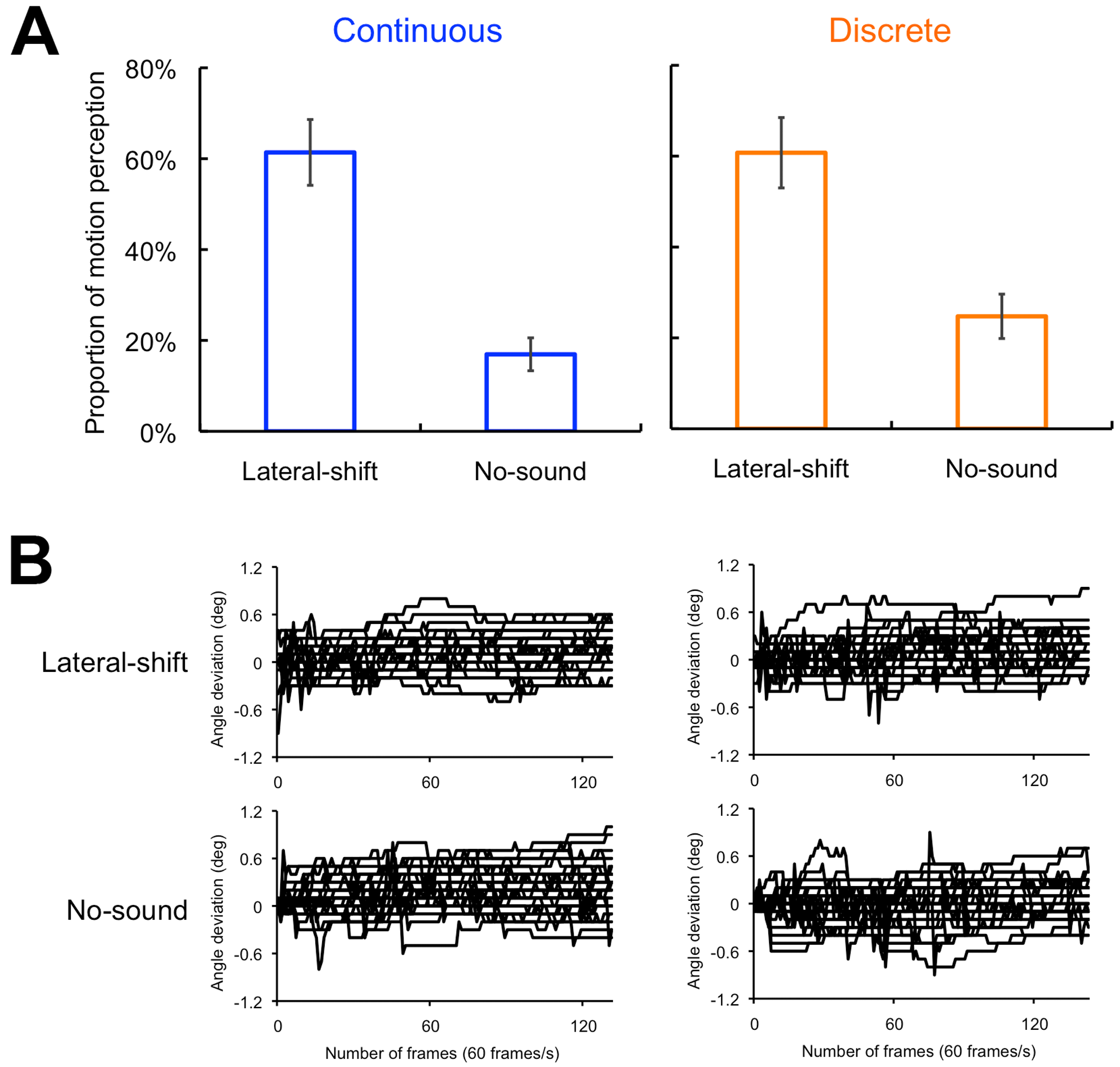

Supplement: Figure S2 — Eye movement data. We conducted a control experiment of Experiment 1 in which eye movements were recorded (continuous block). We also collected data for the discrete sounds (discrete block) (see Figure 4). The lateral-shift and no-sound conditions were presented as the auditory stimuli. The visual stimuli were presented at 10 deg of retinal eccentricity. Each block consisted of 80 trials of the main session with a static flash (auditory conditions (2)×repetitions (40)) and 32 trials of the filler session with a moving (0.2 deg) flash (auditory conditions (2)×repetitions (16)). The participant's eye position was recorded from the left eye at a sampling rate of 60 Hz with EMR-9 (NAC Image Technology, Inc.). Except for these variations, the stimulus parameters and procedures were identical to those of Experiment 1 or the additional experiment for discrete sounds. Trials in which eye position deviated by more than 1 deg of visual angle in the horizontal direction from the center of the fixation point during the stimulus presentation were discarded from the analysis. Whereas 12.1±4.8 (SEM) % and 9.2±3.0 (SEM) % of trials were excluded in the continuous block, 12.1±5.6 (SEM) % and 21.1±6.4 (SEM) % of trials were excluded in the discrete block in each auditory condition (lateral-shift and no-sound), respectively. (A) Proportion of visual motion perception without eye movements (N = 6, including 2 of the authors (S.H. and W.T.)). The error bars denote the standard error of the means. A paired two-tailed t test confirmed that the reliable amount of motion perception occurred in the lateral-shift condition in each block (continuous block: t(5) = 5.25, p<.005; discrete block: t(5) = 3.11, p<.05). We, therefore, could assume that eye movement was not a decisive factor of the result for SIVM. (B) Examples of eye movement recording data for a participant. The upper and lower data show the time course of eye position for the lateral-shift and no-sound conditions in each block, res [file pone.0017499.s002.tif]
